# Predicting blood-to-plasma concentration ratios of drugs from chemical structures and volumes of distribution in humans

**DOI:** 10.1007/s11030-021-10186-7

**Published:** 2021-02-10

**Authors:** Hideaki Mamada, Kazuhiko Iwamoto, Yukihiro Nomura, Yoshihiro Uesawa

**Affiliations:** 1 Department of Medical Molecular Informatics, Meiji Pharmaceutical University, 2-522-1Kiyose-shi, Tokyo, 204-858 Noshio Japan; 2 Drug Metabolism and Pharmacokinetics Research Laboratories, Central Pharmaceutical Research Institute, Japan Tobacco Inc, 1-1, Murasaki-cho, Takatsuki, 569-1125 Osaka Japan

**Keywords:** Blood-to-plasma ratio, Pharmacokinetics, Quantitative structure–pharmacokinetic relationships, Artificial neural networks, Volume of distribution

## Abstract

**Abstract:**

Despite their importance in determining the dosing regimen of drugs in the clinic, only a few studies have investigated methods for predicting blood-to-plasma concentration ratios (Rb). This study established an Rb prediction model incorporating typical human pharmacokinetics (PK) parameters. Experimental Rb values were compiled for 289 compounds, offering reliable predictions by expanding the applicability domain. Notably, it is the largest list of Rb values reported so far. Subsequently, human PK parameters calculated from plasma drug concentrations, including the volume of distribution (Vd), clearance, mean residence time, and plasma protein binding rate, as well as 2702 kinds of molecular descriptors, were used to construct quantitative structure–PK relationship models for Rb. Among the evaluated PK parameters, logVd correlated best with Rb (correlation coefficient of 0.47). Thus, in addition to molecular descriptors selected by XGBoost, logVd was employed to construct the prediction models. Among the analyzed algorithms, artificial neural networks gave the best results. Following optimization using six molecular descriptors and logVd, the model exhibited a correlation coefficient of 0.64 and a root-mean-square error of 0.205, which were superior to those previously reported for other Rb prediction methods. Since Vd values and chemical structures are known for most medications, the Rb prediction model described herein is expected to be valuable in clinical settings.

**Graphical abstract:**

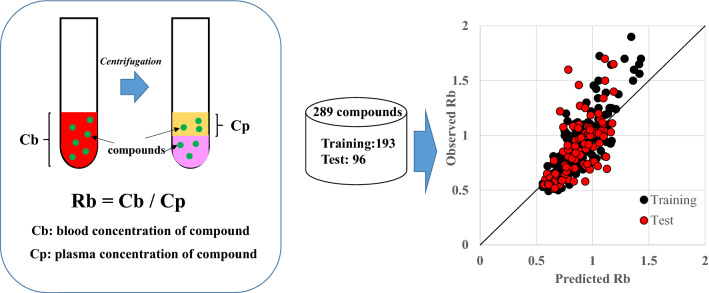

**Supplementary informations:**

The online version of this article (10.1007/s11030-021-10186-7) contains supplementary material, which is available to authorized users.

## Introduction

Blood-to-plasma ratio (Rb) is defined as Cb (blood concentration of compound) / Cp (plasma concentration of compound). It is an important clinical parameter for calculating pharmacokinetic (PK) parameters based on blood concentrations from those based on plasma concentrations. For example, in the case of doripenem, which shows Rb value of 0.5 due to almost no distribution in blood cells, doripenem exposure calculated from plasma is twice the exposure calculated from blood [1]. By contrast, in the case of butorphanol, which shows Rb value of approximately 2 to be concentrated in blood cells, butorphanol exposure calculated from plasma is half the exposure calculated from blood [2]. Therefore, exposure-related PK parameters such as clearance (CL) can deviate significantly from their blood-based and plasma-based values, depending on the Rb. To estimate these PK parameters accurately and determine the suitable dosing regimen in drug therapy, the use of these PK parameters calculated from drug concentrations in the blood is important. However, the PK parameters are typically calculated from plasma drug concentrations rather than blood. For precise PK calculations, these parameters must be converted to blood-derived values by Rb. Regrettably, the number of drugs for which experimental Rb values have been reported is limited; therefore, clinicians must often make a rough approximation, e.g., by assuming that Rb = 1 [3, 4]. However, this assumption's rationale is not clear, and the analyses using this value can be inaccurate.

Quantitative structure–pharmacokinetic relationships (QSPkR) are mathematical approaches for predicting PK parameters based on molecular structures. A number of examples of QSPkR methods have been reported for predicting human PK parameters (e.g., CL, Vd, mean residence times [MRT]) and in vitro parameters (e.g., solubility, metabolic stability, plasma protein binding [PPB]) [5–12]. Determination of Rb is challenging as it involves consideration of the relationships between multiple protein factors, such as the ones between plasma proteins and blood cell binding [13]. QSPkR is a useful approach for modeling these complex relationships and predicting Rb. However, to the best of our knowledge, to date, only one study involved the prediction of human Rb based on QSPkR. Paixão et al. used a dataset of 93 drugs for constructing Rb prediction models based on artificial neural networks (ANN) [14]. They established a regression model with 74 compounds and subsequently evaluated the prediction model using 19 compounds. However, the number of employed compounds was lower than in other QSPkR models [6, 8, 11, 12, 15]. Thus, the available chemical space might be limited. In this study, we used 289 compounds for model construction and evaluation. Notably, this is the largest dataset for Rb prediction reported to date. In addition to PPB used by Paixão et al., there are other important clinical PK parameters, including Vd, CL, and MRT. These clinical PK parameters are available for almost all medications used in the clinic. Because these clinical PK parameters calculated from plasma drug concentrations are Rb-dependent variables, they could be used to predict Rb. The aim of the current study was to build an accurate Rb prediction model to enable precise dosage determination during drug therapy. To achieve this, we attempted to improve the regression model for Rb using 289 compounds and human PK parameters along with molecular descriptors.

## Methods

### Data collection and handling

To create the database, we collected human Rb and PPB data from an in-house database as well as from various articles. Additionally, human intravenous CL and Vd data were collected from previous publications and are summarized in Tables S1 and S2. To select PK parameters for Rb prediction models, a complete dataset of 270 compounds (Rb, PPB, Vd, CL, and MRT) was prepared (Table S1). In addition, we obtained data of 20 compounds, (Supplementary Table S2) for building Rb models (Table S2). In total, 289 compounds were included, while carboplatin was excluded from model building (refer to the “calculation of chemical descriptors” section). According to a previous study, in this work, we used compounds with Rb of < 2.0 [16]. Logarithmically transformed (log) Rb values were used for the prediction.

### Calculation of chemical descriptors

The structural data for each drug were obtained from PubChem (PubChem, https://pubchem.ncbi.nlm.nih.gov/) and DRUGBANK (DRUGBANK, https://www.drugbank.ca/) (Tables S1 and S2). The structural data for water molecules and counter ions were eliminated by processing of disposal salts. Subsequently, the 3D structure of each drug was optimized using “Rebuild 3D,” and the force field calculations (amber-10: EHT) were conducted in Molecular Operating Environment (MOE) version 2018.0101 (MOLSIS Inc., Tokyo, Japan). Structural descriptors were calculated employing MOE and Dragon 7.0 (Kode Srl., Pisa, Italy). Because some descriptors of carboplatin, which contains platinum, could not be calculated, the drug was excluded from building the Rb prediction models. Overlapped and highly correlated (R > 0.99) descriptors as well as those with constant or missing values were removed. In total, 1777 descriptors were selected for further analysis.

### Measurement of PPB

Pooled human plasma was obtained from heparinized blood from nine to ten healthy volunteers not medicated for at least seven days. The human blood collection and PPB experimental protocols were approved by the Ethical Review Committee of Japan Tobacco Inc, Central Pharmaceutical Research Institute. Informed consent was obtained from all subjects, and the PPB experiments were conducted in accordance with the Ethical Guidelines for Medical and Health Research Involving Human Subjects. Binding of compounds to plasma protein was evaluated by equilibrium dialysis utilizing a 96-well equilibrium dialysis unit and HTD96b dialysis membrane strips with a molecular cutoff of 12–14 kDa (HTDialysis, Gales Ferry, CT, USA). Plasma containing the compounds (final concentration of 5 µM) and buffer (Dulbecco’s phosphate-buffered saline; Thermo Fisher Scientific, Waltham, MA) were added into the donor and receiver cells, respectively. Following dialysis for 5 h at 37 °C, the concentrations of the compounds in the plasma and buffer were determined by liquid chromatography–tandem mass spectrometry (LC–MS/MS) (Acquity™ Ultra Performance LC I-Class and Xevo TQ-S; Waters, Milford, MA). The percentage of free fraction (fu) was calculated according to the following equation [17]:

1 $$\mathrm{Free fraction }(\mathrm{fu},\mathrm{ \%})=\frac{\mathrm{Concentration of buffer}}{\mathrm{Concentration of plasma}}\times 100$$

### Measurement of Rb

Heparinized blood was collected from three to seven healthy volunteers not medicated for at least seven days. Human blood collection and Rb experimental protocols were approved by the Ethical Review Committee of Japan Tobacco Inc, Central Pharmaceutical Research Institute. Informed consent was obtained from all subjects and the Rb experiments were conducted in accordance with the Ethical Guidelines for Medical and Health Research Involving Human Subjects. The blood was spiked with the compounds (final concentration of 5 µM) and incubated at 37 °C for 30 min. Following incubation, the samples were centrifuged at 5000 rpm for 5 min to obtain plasma. Subsequently, the concentrations of the compounds in the plasma were determined by LC–MS/MS using the same method as described for the measurement of PPB. Rb was calculated according to Eq. (2). The measured hematocrit values ranged from 0.45 to 0.46.

2 $$\mathrm{Rb}=\frac{\mathrm{Initial blood concentration}}{\mathrm{Drug concentration in plasma}}$$

### Calculation of MRT

The MRT values of the compounds were calculated by a noncompartment model using the following equation [18]:

3 $$\mathrm{MRT }(\mathrm{hr})=\frac{Vd(L/kg)}{CL(L/hr/kg)}$$

### Selection of PK parameters for Rb prediction models

To select appropriate PK parameters for the Rb prediction models, correlation analysis was performed using correlation coefficients between log human Rb and the PK parameters for selection (original or log) employing the JMP® Pro software 14.3.0 (SAS Institute Inc., Cary, NC, USA). PK parameters were chosen based on the R values.

### Selection of molecular descriptors

We reduced the size of descriptors because 1777 molecular descriptors are very large compared to the training data and reduce the amount of calculation. To select an algorithm for calculating the importance of descriptors, Rb prediction models were constructed using logVd and 1777 different molecular descriptors. Pipeline Pilot 2019 RRID:SCR_014917 (DASSULT SYSTEMS, https://www.3dsbiovia.com/products/collaborative-science/biovia-pipeline-pilot/) was employed to build Rb models using support vector regression (SVR), random forest, XGBoost, and genetic algorithm–multiple linear regression (GA-MLR) with hyper-parameters (Table S3 a–d). Following fivefold cross-validation, the algorithms were selected based on the results of internal validation using R values and root-mean-square errors (RMSE [log]) (4) as evaluation scores.

4 $$\mathrm{RMSE}(\mathrm{Log})=\sqrt{\frac{\sum {(\mathrm{log predicted}-\mathrm{log observed})}^{2}}{n}}$$

For the molecular descriptors selection, Gain, an index of importance for XGBoost, was calculated. After calculating Gain, logVd and 140 molecular descriptors were selected, and the number of molecular descriptors was then reduced to 100 based on this Gain.

### Separation of compounds into training and test sets and their verification by chemical space analysis

After sorting based on Rb, the compounds in the dataset were separated randomly into a training set and a test set at a ratio of 2:1. To investigate applicability domain, 11 molecular parameters were used as reported previously [19] with JMP Pro PCA (Principal component analysis) [19]. The parameters included molecular weight, SlogP (log octanol/water partition coefficient), topological polar surface area (TPSA), h_logD (octanol/water distribution coefficient [pH = 7]), h_pKa (acidity [pH = 7]), h_pKb (basicity [pH = 7]), a_acc (number of H-bond acceptor atoms), a_don (number of H-bond donor atoms), a_aro (number of aromatic atoms), b_ar (number of aromatic bonds), and b_rotN (number of rotatable bonds). The principal components were calculated from 1 to 3.

### Construction of Rb models

Pipeline Pilot 2019 was employed to construct the Rb models using SVR, random forest, XGBoost, and GA-MLR with hyper-parameters (Table S3 a–d). JMP Pro was used to construct the Rb models using ANN with hyper-parameters (Table S3 e). Ten models with different random seeds were constructed, and the mean of predicted value was calculated. Evaluation scores were calculated based on the mean of predicted value. In each model, logVd and 100 molecular descriptors, which were selected based on Gain calculated by XGBoost, were used. For all algorithms, fivefold cross-validation was implemented.

### Investigation of the effect of incorporation of Vd on the performance of the models

To establish the impact of Vd on the performance of the models, models based on SVR, random forest, XGBoost, GA-MLR, and ANN were constructed with and without Vd. Moreover, the effect of reducing the number of molecular descriptors was examined using ANN. Ten models with different random seeds were constructed and the mean of predicted value was calculated. Evaluation scores were calculated based on the mean of predicted value.

### Assessment of prediction accuracy

The correlation between the predicted and observed values was determined based on the correlation coefficient (R). The predictability of the models with respect to individual drugs was evaluated based on the fold error calculated using the following equations:

5 $$\mathrm{Fold error}= \frac{\mathrm{predicted value}}{\mathrm{observed value}} (\mathrm{predicted}>\mathrm{observed})$$

6 $$\mathrm{Fold error}= \frac{\mathrm{observed value}}{\mathrm{predicted value}} (\mathrm{observed}>\mathrm{predicted})$$

The overall predictability of each model was assessed using the average fold error (AFE), RMSE, and the mean absolute error (MAE), which were calculated according to the following equations:

7 $$\mathrm{AFE}={10}^{\frac{\sum \mathrm{logfold error}}{n}}$$

8 $$\mathrm{RMSE}=\sqrt{\frac{\sum {(\mathrm{predicted}-\mathrm{observed})}^{2}}{n}}$$

9 $$\mathrm{MAE}=\frac{\sum \left|\mathrm{predicted}-\mathrm{observed}\right|}{n}$$

where n refers to the number of evaluated compounds.

Moreover, percentage of values within a 1.25-fold change was also determined for comparative assessment of predictability.

## Results

### PK parameters selected for constructing Rb models

The constructed dataset included human PPB, CL, Vd, MRT, and Rb. Both logarithmic and original values of all parameters were considered. Notably, the dataset of 270 compounds with no missing values of the selected PK parameters and logVd gave the best result (R = 0.47) in the correlation analysis with a statistically significant difference (Table 1, Fig. S1). This indicated that logVd was the most important among the four evaluated parameters.

**Table 1:** Correlations between Rb and 4 pharmacokinetics (PK) parameters

PK parameter	Transformation	R	*p*-value
Vd	log	0.47	< 0.0001
fp	log	0.42	< 0.0001
fp	–	0.35	< 0.0001
CL	log	0.30	< 0.0001
CL	–	0.25	< 0.0001
Vd	–	0.25	< 0.0001
MRT	log	0.16	0.008
MRT	–	0.03	0.5939

(Logarithmically [log] transformed or not [-]) (*n*  =  270)

Clearance (CL), volume of distribution (Vd), mean resistance time (MRT), free fraction plasma protein binding (fp)

*p*-values were calculated based on analysis of variance

### Selection of molecular descriptors

A dataset containing logRb, logVd, and 1777 different molecular descriptors was constructed. To select an algorithm for the calculation of importance of molecular descriptors, models using SVR, random forest, XGBoost, and GA-MLR were built employing the dataset, and fivefold cross-validation was applied. The internal validation results are summarized in Table 2. Since XGBoost exhibited the best score (R = 0.615 and RMSE (log) = 0.102) among 4 algorithms, it was chosen for the calculation of the importance of 1777 molecular descriptors. LogVd and 140 molecular descriptors were selected based on gain calculated by XGBoost (Table S4).

**Table 2:** Comparison of algorisms for selection of molecular descriptors in the internal validation set

	Random Forest	XGBoost	SVR	GA-MLR
R^a^	0.592	0.615	0.531	0.579
RMSE (log)^b^	0.105	0.102	0.110	0.106

^a^R and RMSE were calculated using logarithmically transformed human Rb

^b^RMSE were calculated using Eq. (4)

### Separation of compounds into training and test sets and their verification by chemical space analysis

**Fig. 1:**
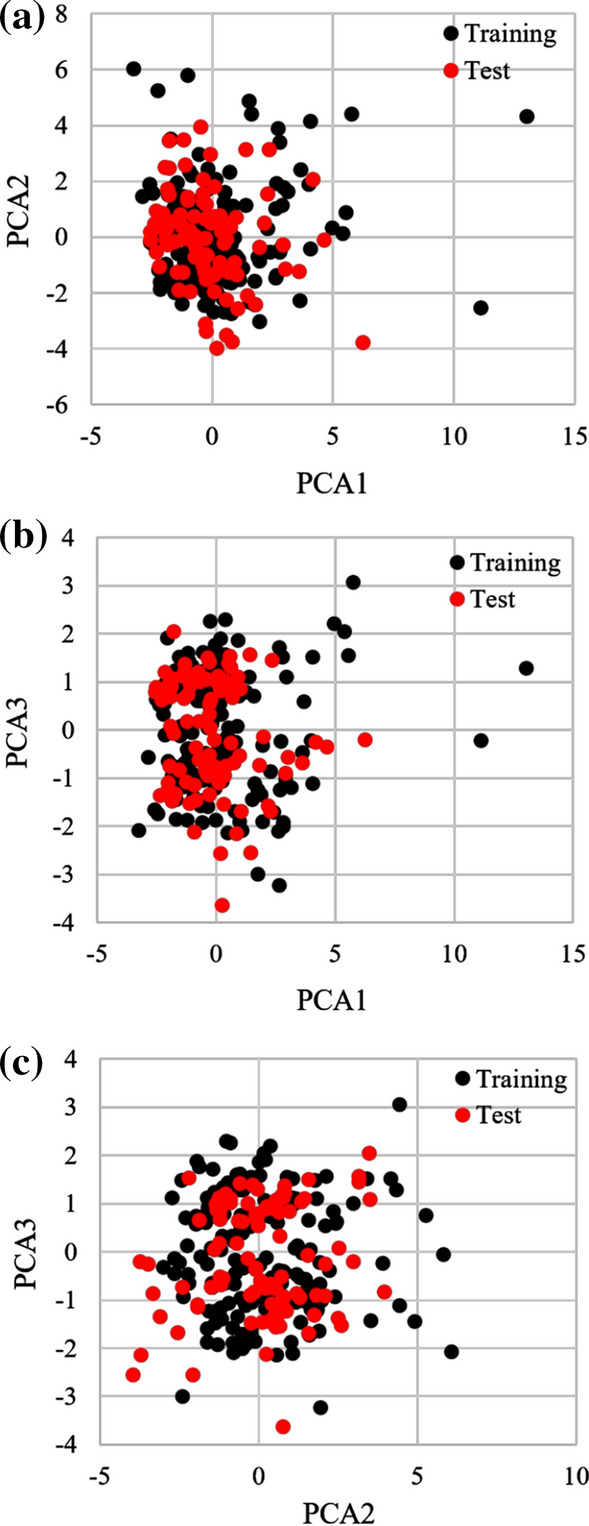
Three-component principal component analysis (PCA) score plots based on 11 representative molecular descriptors (*n* = 289). **a** Score plot of PCA1 (34.4%) and PCA2 (26.1%). The horizontal axis indicates the first principal component, while the vertical axis refers to the second principal component. **b** Score plot of PCA1 (34.4%) and PCA3 (12.5%). The horizontal axis indicates the first principal component, while the vertical axis refers to the third principal component. **c** Score plot of PCA2 (26.1%) and PCA3 (12.5%). The horizontal axis indicates the second principal component, while the vertical axis refers to the third principal component. Each dot represents a compound; black circle is the training set (*n* = 193), whereas the red circle is the test set (*n* = 96)

Principal component analysis (PCA) was performed using a dataset of 289 compounds with 11 representative molecular descriptors to confirm the correctness of the compound separation. It was previously reported that PCA could show an applicability domain [20]. Component one, two, and three explained 34.4%, 26.1%, and 12.5% of the variance, respectively. Figure 1 suggests that the compounds were effectively separated into the training and test sets.

### Construction of the Rb prediction models using Vd and 100 selected molecular descriptors

The Rb prediction models were constructed using logVd and selected top 100 molecular descriptors (Table S4) by SVR, random forest, XGBoost, GA-MLR, and ANN. Table 3 shows the evaluation scores of the external validation set for each algorithm with original Rb. RMSE was calculated according to Eq. (8). The results demonstrated that ANN was the best algorithm for the Rb prediction model among 5 algorithms, and the scores were as follows: RMSE = 0.213, R = 0.605, AFE = 1.186, %inside 1.25-fold = 71.9%, and MAE = 0.158.

**Table 3:** Evaluation of effect of incorporation of volume of distribution (Vd) (with Vd) in the external validation set

	ANN	Random forest	XGBoost	SVR	GA-MLR
RMSE	0.213	0.221	0.218	0.216	0.222
R	0.605	0.562	0.578	0.5989	0.559
AFE	1.186	1.191	1.190	1.197	1.189
% inside 1.25-fold	71.9	70.8	68.8	67.7	72.9
MAE	0.158	0.159	0.159	0.160	0.156

Original Rb values were used

The number of molecular descriptors: 100

The ANN evaluation scores were calculated from the average of each predicted value calculated by 10 different random seed conditions

### Construction of the Rb prediction models using 100 selected molecular descriptors

To examine the effect of incorporation of Vd, we constructed the Rb models without this parameter using SVR, random forest, XGBoost, GA-MLR, and ANN. For ANN, the evaluation scores of the external validation set were as follows: RMSE = 0.226, R = 0.537, AFE = 1.198, %inside 1.25-fold = 67.7%, and MAE = 0.165. These outcomes suggested that the Rb prediction models using Vd were better than those without the parameter. Other models showed comparable results (Table 4).

**Table 4:** Evaluation of the effect of incorporation of volume of distribution (Vd) (without Vd) in the external validation set

	ANN	Random forest	XGBoost	SVR	GA-MLR
RMSE	0.226	0.230	0.238	0.223	0.241
R	0.537	0.511	0.460	0.552	0.433
AFE	1.198	1.198	1.212	1.205	1.225
% inside 1.25-fold	67.7	67.7	64.6	67.7	59.4
MAE	0.165	0.164	0.176	0.166	0.181

Original Rb values were used

The number of molecular descriptors: 100

The ANN evaluation scores were calculated from the average of each predicted value calculated by 10 different random seed conditions

### Optimization of the number of molecular descriptors

**Fig. 2:**
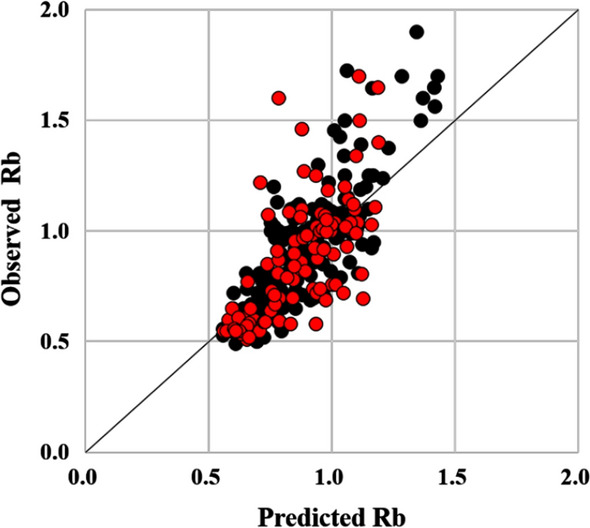
Scatter plot of the training and test sets. The horizontal axis indicates the predicted Rb, while the vertical axis refers to the observed Rb. Each dot represents a compound; black circle is the training set (*n* = 193), whereas the red circle is the test set (*n* = 96). The solid line represents unity

The number of molecular descriptors was optimized by gradually reducing it from 50 to 3 with or without Vd using ANN. Table 5 shows the evaluation scores of the external validation results in the optimization process. The results indicated that the Rb prediction models with Vd were better than those without Vd. The Rb prediction model with Vd and six molecular descriptors exhibited RMSE = 0.205, R = 0.641, AFE = 1.170, %inside 1.25-fold = 74.0%, and MAE = 0.144 (Fig. 2). Notably, these scores were better than those for the model with Vd and 100 molecular descriptors.

**Table 5:** External validation results in optimization process of the number of molecular descriptors

Number of MD	50		25		12		6		3	
Human Vd	+	–	+	–	+	–	+	–	+	–
RMSE	0.216	0.235	0.213	0.227	0.213	0.237	0.205	0.231	0.224	0.243
R	0.589	0.480	0.607	0.532	0.605	0.465	0.641	0.506	0.547	0.415
AFE	1.189	1.209	1.186	1.205	1.177	1.209	1.170	1.208	1.174	1.208
% inside 1.25-fold	64.6	65.6	67.7	69.8	74.0	62.5	74.0	64.6	72.9	68.8
MAE	0.161	0.172	0.158	0.168	0.150	0.172	0.144	0.169	0.147	0.171

MD: molecular descriptor

The evaluation scores were calculated from the average of each predicted value calculated by 10 different random seed conditions

## Discussion

In the clinic, Rb is an important variable for the calculation of PK parameters based on blood concentrations from those based on plasma concentrations. When Rb of a compound is not available, it is generally assumed to be 1. Other assumptions consider ionization of compounds, i.e., Rb is assumed to be 1 for neutral and basic compounds, while for acidic compounds Rb = 0.55 [21, 22]. Nonetheless, this assumption is occasionally inaccurate; therefore, the development of Rb prediction methods optimized for each compound is essential. One study described an Rb prediction model constructed using 74 compounds for train, 19 compounds for internal validation, 7 compounds for external validation, human PPB data, and ten molecular descriptors based on ANN [14]. However, the number of compounds is small and does not fill a large chemical space. Also, due to the small number of external validation, it has not been sufficiently validated. For this reason, we acquired experimental data and increased training data. Hence, in the current study, we built Rb prediction models using a larger number of compounds and investigated the correlation between various PK parameters and Rb. This model may be a more reliable prediction model than the previous one because it covers a larger chemical space, which is considered a limitation of the current condition. According to previous reports, compounds with Rb < 2 were included in the analysis [14, 16].

Due to their availability, we selected in vivo human PK parameters (i.e., CL, Vd, and MRT) for the Rb prediction models. Moreover, as Paixão selected PPB for their Rb prediction model, the parameter was also included in the analysis conducted herein. Correlation analysis was performed between Rb and four parameters (i.e., PPB, CL, Vd, and MRT). Among these parameters, logVd showed the highest correlation (R = 0.47) (Table 1, Fig. S1). Thus, we selected logVd for the construction of the Rb prediction models. Some reports indicated that Rb might be related to Vd. Hinderling found that unbound in vivo steady-state Vd correlated with in vitro red blood cells in terms of the plasma water partition coefficients [13]. It is known that acidic phospholipids are components of the plasma membrane and bind to basic compounds. It is noteworthy that the amount of acidic phospholipids in the muscle is similar to that in the blood cells [23]. Since the muscle is one of the tissues with the largest volume in humans, the relationship between Rb and Vd was suggested in previous studies [23, 24]. Rodgers reported that the electrostatic interactions between drugs and blood cell acidic phospholipids must be considered to improve the prediction accuracy of drug distribution to organs [25]. It was suggested that Rb was related to Vd of basic drugs. In contrast, acidic compounds tend to bind to albumin, which is the most abundant plasma component. Thus, it is considered that the PPB affects Rb and Vd. These findings also indicate that Vd might be related to Rb both in the case of basic and acidic compounds. Notably, this study is the first to use Vd for Rb prediction.

We used MOE and Dragon software to calculate the molecular descriptors. Consequently, 2702 molecular descriptors were calculated for each compound (Dragon: 2185 descriptors [descriptors with constant values were excluded], MOE: 517 descriptors). Because the number of descriptors was larger than the number of compounds, we screened out a selection of the descriptors. In the first instance, we excluded descriptors with constant or missing values as well as those highly correlated (R > 0.99) to other descriptors. In total, 1777 descriptors were selected. We subsequently constructed Rb prediction models using logVd and 1777 descriptors. Algorithms were selected for the calculation of the importance of molecular descriptors. Based on the internal validation results, we selected XGBoost, which exhibited the highest R (0.615) and the lowest RMSE (log) (0.102) (Table 2). XGBoost is considered as a valuable algorithm [26], which can be employed for the construction of prediction models and visualization of the importance of variables. In this work, logVd and 140 molecular descriptors with high importance (determined based on Gain) were selected by XGBoost. Intriguingly, logVd was selected as the most important parameter for the Rb prediction. We then selected the top 100 important molecular descriptors to construct the Rb models (Table S4).

**Fig. 3:**
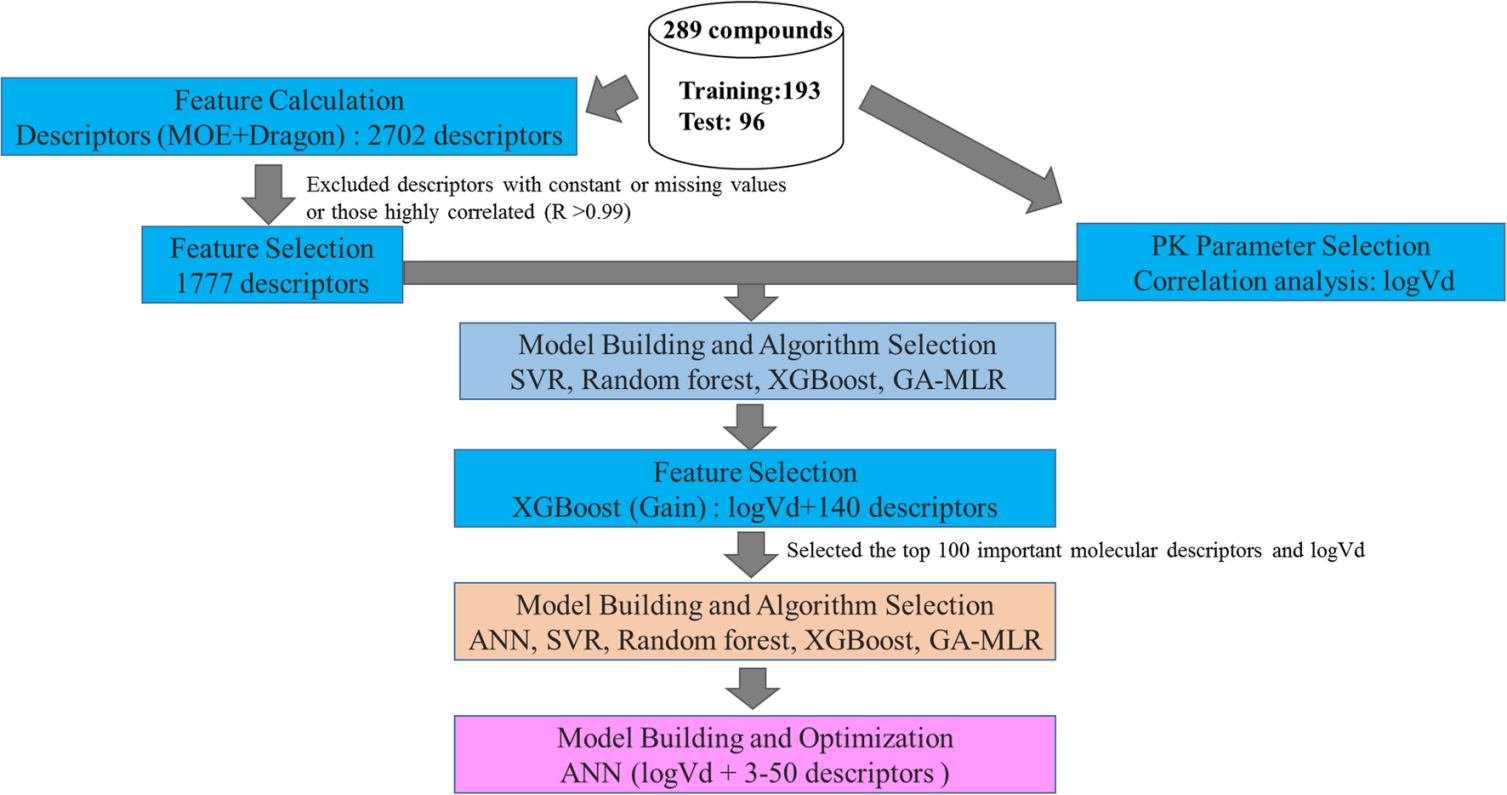
Flowchart of the modeling process for Rb prediction

The compounds were separated into the training and test sets for the construction and subsequent verification of the prediction models. To ensure unbiased segregation, the PCA analysis was conducted based on 11 representative molecular descriptors, which are generally considered important for synthetic expansion [19, 20]. As shown in Fig. 1, the separation was well balanced and the cumulative contribution ratio of PCA from 1 to 3 was 72.6%. These results showed similar trends when PCA was performed with 100 descriptors in the model (Fig. 3S).

To select suitable algorithms for the Rb prediction models, the models were constructed using SVR, random forest, XGBoost, GA-MLR, and ANN with logVd and 100 selected molecular descriptors. ANN showed the best score (R = 0.605 and RMSE = 0.213) among 4 algorithms in the external validation set (Table 3). Interestingly, ANN was also selected for Rb prediction in a previous report [14]. To evaluate the effect of Vd, we constructed prediction models using 100 descriptors without logVd (Table 4). All evaluation scores became worse, implying that Vd was important parameter to construct accurate Rb prediction models.

Furthermore, to construct Rb prediction models using fewer descriptors, we reduced the number of molecular descriptors from 100 to 3. Pleasingly, as demonstrated in Table 5, the Rb prediction model with logVd and six molecular descriptors exhibited similar or better scores (R = 0.641 and RMSE = 0.205) to that of a model using logVd and 100 descriptors in the external validation set (Fig. 2). Uchimura reported an Rb prediction method using rat Rb and human PPB with 58 compounds, which showed an R value of 0.603 [16]. The model developed in this study using the test set (96 compounds) did not contain any Rb-related data and showed almost the same R value (0.641) as Uchimura’s model. Paixão determined that in their Rb model, the percentage of predicted values within a 1.25-fold limit was 84% (out of 19 compounds) [14]. However, Paixão’s group used early stopping based on the RMSE of the test set, indicating that their test set might be an internal validation set. Paixão also performed external validation; however, the validation set consisted of just seven compounds [14]. In contrast, our test set was an external validation set, which contained a larger number of compounds. Hence, accurate comparison of our results with the outcomes of other studies was challenging. In this work, we constructed an Rb model with Vd and six molecular descriptors by ANN based on a flow chart of the model construction (Fig. 3). Importantly, this Rb model was better than that obtained by assuming Rb = 1 (RMSE = 0.288), and the number of descriptors in our model was lower than in Paixão’s Rb model (PPB and ten molecular descriptors).

As shown in Table 6, six molecular descriptors were selected in the final Rb prediction model. Among these six molecular descriptors, MATS1i and ASA- were related to compound ionization or partial charge. Paixão et al. also used ionization-related parameters, including pKabase and pKaacid [14]. Hence, molecular descriptors related to compound ionization or partial charge were selected in both models. Since mainly non-ionized compounds can penetrate blood cells, it is considered that Rb is affected by compound ionization and partial charge. In addition to compound ionization parameters, our model contained lipophilicity parameters (e.g., SlogP_VSA9). The Rb model developed by Paixão et al. did not consider LogP/D-related parameters, and PPB was used instead. Because PPB is related to LogD/P [27, 28], PPB might include LogP/D effects. Fagerholm investigated the correlation between LogD and Rb using 48 compounds and established the lack of direct correlation between them [29]. Accordingly, Uchimura reported similar results [16]. In the present study, no direct correlation was found between SlogP_VSA9 and Rb (Fig. S2). However, SlogP_VSA9 was selected by XGBoost based on the importance (Gain) for Rb prediction. Since XGBoost is a non-linear algorithm, the existence of an indirect correlation between LogP/D and Rb was suggested. Rb is related not only to the penetration into blood cells but also to PPB. Moreover, both processes are related to LogP/D [30]. Although no direct correlation between Rb and LogP/D was indicated, the existence of a more complex correlation between the parameters is possible.

**Table 6:** Details of 6 molecular descriptors in Rb prediction models

Descriptor	Software to calculate molecular descriptor	Descriptions
ASA-	MOE	Descriptor related electrostatic properties
pmi	MOE	Principal moment of inertia
h_logS	MOE	Log solubility in water
SlogP_VSA9	MOE	Descriptor related LogP and molecular size
MATS1i	Dragon	Descriptor related electrostatic properties
h_pstates	MOE	The entropic count or fractional number of protonation states

In clinical practice involving drug treatment, the human Vd values of compounds are generally known. Nevertheless, as previously mentioned, since the experimental values of Rb are often unknown, to predict drug blood levels, Rb is often assumed to be 1 or 0.55 [3, 4, 31, 32]. Such assumptions are typically made in the prediction of the effect of blood concentration on drug–drug interactions, population PK, and special populations. As some compounds exhibit a narrow safety margin, accurate prediction of blood concentrations is important to prevent the occurrence of adverse effects. Instead, of using constants, appropriate Rb values should be considered for each drug. The Rb prediction method described herein can be employed for the prediction of Rb using six descriptors and human Vd. Thus, our model enables accurate prediction of blood concentrations when experimental Rb values are not available.

## Conclusions

The present study found that Vd is an important parameter for constructing Rb prediction models. An Rb model was constructed using a combination of descriptors (Vd and six molecular descriptors) based on ANN. In a clinical setting, the Vd values of drugs are typically available, while the Rb values are occasionally missing. In such situations, the model developed herein could be employed to estimate Rb and obtain PK parameters based on blood concentrations.

## Supplementary informations

Below is the link to the electronic supplementary material.

Supplementary Dataset

Supplementary Dataset2

Supplementary Information

Supplementary Information_revision_20210112y
