# Supplementary material for: Predicting blood-to-plasma concentration ratios of drugs from chemical structures and volumes of distribution in humans
Source: Mol Divers. 2021 Feb 10;25(3):1261–70. doi: 10.1007/s11030-021-10186-7 (PMC8342319; doi:10.1007/s11030-021-10186-7)
Supplement: Supplementary file 1 — Supplementary Dataset [file 11030_2021_10186_MOESM1_ESM.docx]

**Predicting blood-to-plasma concentration ratios of drugs from the chemical structures and volumes of distribution in humans**

Hideaki Mamada, MS *^1,2^, Kazuhiko Iwamoto, MS ^1^, Yukihiro Nomura, PhD ^1^, Yoshihiro Uesawa, PhD *^2^

1 Drug Metabolism and Pharmacokinetics Research Laboratories, Central Pharmaceutical Research Institute, Japan Tobacco Inc., Osaka, Japan.

2. Department of Medical Molecular Informatics, Meiji Pharmaceutical University, Tokyo, Japan

*Corresponding author.

Phone: +81-42-495-8983; FAX: +81-42-495-8983;

E-mail address: uesawa@my-pharm.ac.jp (Y.U.)

**Supporting Information 1**

Supporting Table (file type; Excel)

Tabular. Table S1. Blood-to-plasma concentration ratio and PK parameters for the 270 drugs.

Tabular. Table S2. Blood-to-plasma concentration ratio and Vd for the 20 drugs.

Tabular. Table S4. Gain of 141 descriptors

**Supporting Information 2**

Supporting Tables and Figures (Table SIV, Figures S1-S2) (file type;word)

Table S3-a. Parameter ranges for SVR

Table S3-b. Parameter ranges for random forest

Table S3-c. Parameter ranges for XGBoost

Table S3-d. Parameter ranges for GA-MLR

Table S3-e. Parameter ranges for ANN

Figure S1. Correlation between Rb and Vd (n=270)

Figure S2. Relationship between SlogP_VSA9 and log Vd. SlogP_VSA9 was calculated based on MOE (n=289).

Figure. S3. Three-component principal component analysis (PCA) score plots based on 100 molecular descriptors (n = 289).

**Table S3-a.** Parameter ranges for SVR

| SVR_GridSearch | | |
| --- | --- | --- |
| Learner Kernel | Radial |  |
| Gamma | 0.1/nx-1/nx |  |
| Cost | 0.01-1000 |  |
| Epsilon | 0.001-1 |  |

**Table S3-b.** Parameter ranges for random forest

| random forest_GridSearch | |
| --- | --- |
| Maximum Tree Depth | 3-100 |
| Number of Trees | 100-3000 |

**Table S3-c.** Parameter ranges for XGBoost

| **XGBoost_GridSearch** | |
| --- | --- |
| Learner Booster | gbtree |
| Max trees | 10-300 |
| Learning Rate | 0.01-0.5 |
| Max Depth | 2-10 |
| Data Fraction | 0.5-1 |
| Descriptor Fraction | 0.05-1 |

**Table S3-d.** Parameter ranges for GA-MLR

| **GA-MLR_GridSearch** | |
| --- | --- |
| Number of models to keep | 5-500 |
| Model form | Linear |
| Mixture Model Form | Linear |
| Maxium Equation Length | 10-100 |
| Learner Population Size | 100-1000 |
| Learner Maximum Generations | 500-2000 |
| Learner LOF Smoothness Parameter | 0.25-0.75 |

**Table S3-e.** Parameter ranges for ANN

| **ANN_GridSearch** | |
| --- | --- |
| Activation Type | TanH |
| Layer | 3 |
| Boosting number of models | 1000 |
| Learning Rate | 0.1 |
| Penalty Method | Squared |


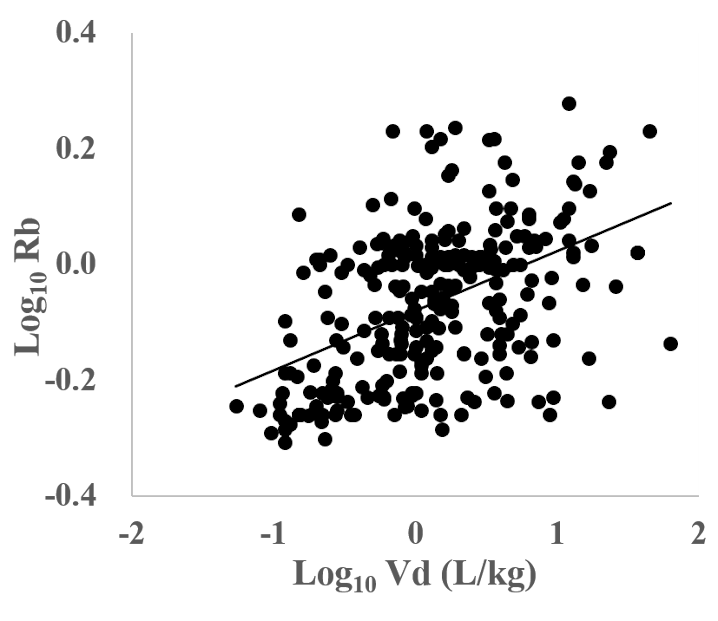


**Figure S1. Correlation between Rb and Vd (n=270)**

The horizontal axis is Log_10_ Vd (L/kg), and the vertical axis is Log_10_ Rb. The solid line represents the regression line.


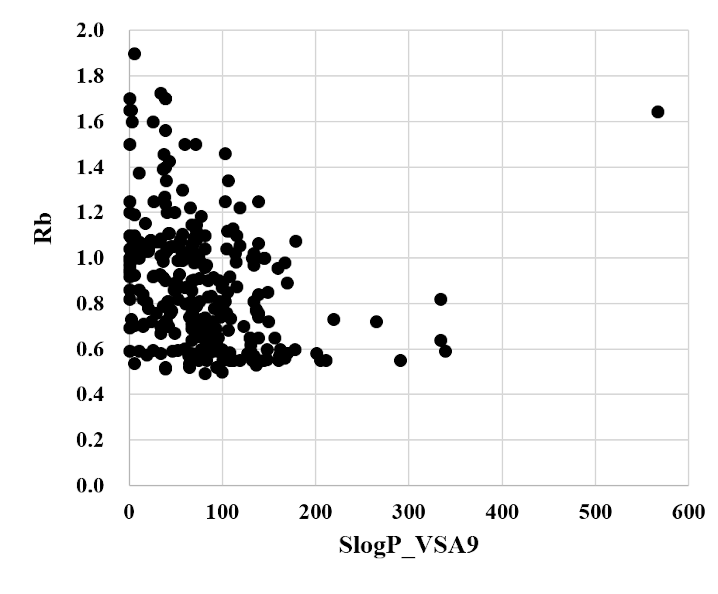


**Figure S2. Relationship between SlogP_VSA9 and log Vd. SlogP_VSA9 was calculated based on MOE (n=289).**


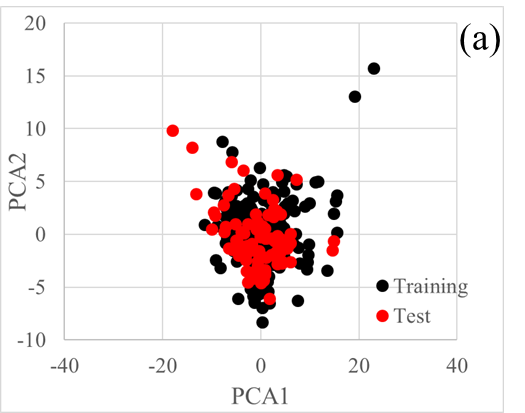


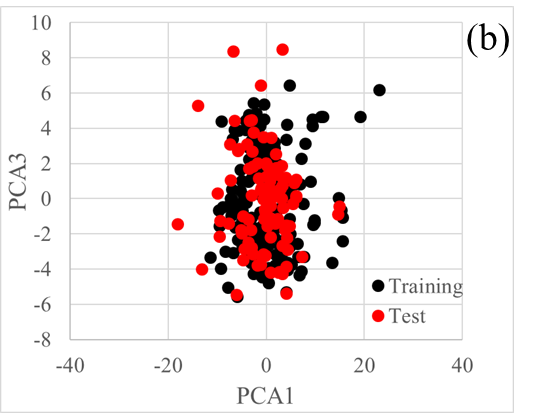


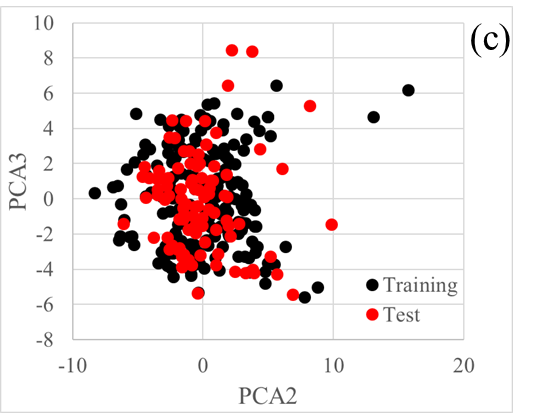


**Figure. S3. Three-component principal component analysis (PCA) score plots based on 100 molecular descriptors (n = 289).** (a) Score plot of PCA1 (28.6%) and PCA2 (9.83%). The horizontal axis indicates the first principal component, while the vertical axis refers to the second principal component. (b) Score plot of PCA1 (28.6%) and PCA3 (7.79%). The horizontal axis indicates the first principal component, while the vertical axis refers to the third principal component. (c) Score plot of PCA2 (9.83%) and PCA3 (7.79%). The horizontal axis indicates the second principal component, while the vertical axis refers to the third principal component. Each dot represents a compound; black circle is the training set (n = 193), whereas the red circle is the test set (n = 96)

References

1. Lombardo, F., Waters, N. J., Argikar, U. A., Dennehy, M. K., Zhan, J., Gunduz, M., Harriman S. P., Berellini G, Liric Rajlic I Obach, R. S. (2013). Comprehensive assessment of human pharmacokinetic prediction based on in vivo animal pharmacokinetic data, part 2: Clearance. *Journal of Clinical Pharmacology*, *53*(2), 178–191. <https://doi.org/10.1177/0091270012440282>
2. Obach, R. S., Lombardo, F., & Waters, N. J. (2008). Trend analysis of a database of intravenous pharmacokinetic parameters in humans for 670 drug compounds. *Drug Metabolism and Disposition*, *36*(7), 1385–1405. <https://doi.org/10.1124/dmd.108.020479>
3. Y. Rakhmanina, N., Dirajlal-Fargo, S., V. Capparelli, E., & Mirochnik, M. (2012). Pharmacokinetic Considerations of Perinatal Antiretroviral Therapy. *Current Drug Metabolism*, *13*(6), 744–759. <https://doi.org/10.2174/138920012800840365>
4. Chiou, W. L., Robbie, G., Chung, S. M., Wu, T. C., & Ma, C. (1998). Correlation of plasma clearance of 54 extensively metabolized drugs between humans and rats: Mean allometric coefficient of 0.66. *Pharmaceutical Research*, Vol. 15, pp. 1474–1479. <https://doi.org/10.1023/A:1011974226596>
5. Zhang, D. Evaluation of the Allometric Exponents in Prediction of Human Drug Clearance. (2014).Virginia Commonwealth University (VCU Scholars Compass) <https://scholarscompass.vcu.edu/etd/3533>
6. Rodgers, T., & Rowland, M. (2007). Mechanistic approaches to volume of distribution predictions: Understanding the processes. Pharmaceutical Research, 24(5), 918–933. <https://doi.org/10.1007/s11095-006-9210-3>
7. Paixão, P., Gouveia, L. F., & Morais, J. A. G. (2010). Prediction of the in vitro intrinsic clearance determined in suspensions of human hepatocytes by using artificial neural networks. *European Journal of Pharmaceutical Sciences*, *39*(5), 310–321. <https://doi.org/10.1016/j.ejps.2009.12.007>
8. Jolivette, L. J., & Ward, K. W. (2005). Extrapolation of human pharmacokinetic parameters from rat, dog, and monkey data: Molecular properties associated with extrapolative success or failure. *Journal of Pharmaceutical Sciences*, *94*(7), 1467–1483. <https://doi.org/10.1002/jps.20373>
9. Fleishaker, J. C., Friedman, H., & Pollock, S. R. (1991). Extent and Variability of the First-Pass Elimination of Adinazolam Mesylate in Healthy Male Volunteers. *Pharmaceutical Research: An Official Journal of the American Association of Pharmaceutical Scientists*. , 8(2), 162–167. <https://doi.org/10.1023/A:1015875516834>
10. Paixão, P., Gouveia, L. F., & Morais, J. A. (2009). Prediction of drug distribution within blood. *European Journal of Pharmaceutical Sciences*, *36*(4–5), 544–554. <https://doi.org/10.1016/j.ejps.2008.12.011>
11. Uchimura, T., Kato, M., Saito, T., & Kinoshita, H. (2010). Prediction of Human Blood-to-Plasma Drug Concentration Ratio. BIOPHARMACEUTICS & DRUG DISPOSITION, 31, 286–297. <https://doi.org/10.1002/bdd>
12. FDA approval package document Lotronex (Alosetron HCL) Company: Glaxo Wellcome Inc. Application No.: 021107 Approval Date: 2/11/2000 Pharmacology Review P25-26 ; 2020 September 6. Available from <https://www.accessdata.fda.gov/drugsatfda_docs/nda/2000/21107a_Lotronex_phrmr_P2.pdf> [Website]
13. Kato, M., Shitara, Y., Sato, H., Yoshisue, K., Hirano, M., Ikeda, T., & Sugiyama, Y. (2008). The quantitative prediction of CYP-mediated drug interaction by physiologically based pharmacokinetic modeling. *Pharmaceutical Research*, *25*(8), 1891–1901. <https://doi.org/10.1007/s11095-008-9607-2>
14. Berry, L. M., Li, C., & Zhao, Z. (2011). Species Differences in Distribution and Prediction of Human Vss from Preclinical Data. *Drug Metabolism and Disposition*, *39*(11), 2103 – 2116. <https://doi.org/10.1124/dmd.111.040766.for>
15. Akabane, T., Tabata, K., Kadono, K., Sakuda, S., Terashita, S., & Teramura, T. (2010). A Comparison of Pharmacokinetics between Humans and Monkeys. *Drug Metabolism and Disposition*, *38*(2), 308–316. <https://doi.org/10.1124/dmd.109.028829.Fagerholm>
16. Small, H., Gardner, I., Jones, H. M., Davis, J., & Rowland, M. (2011). Measurement of binding of basic drugs to acidic phospholipids using surface plasmon resonance and incorporation of the data into mechanistic tissue composition equations to predict steady-state volume of distribution. *Drug Metabolism and Disposition*, *39*(10), 1789–1793. <https://doi.org/10.1124/dmd.111.040253>
17. Miyamoto, M., Iwasaki, S., Chisaki, I., Nakagawa, S., Amano, N., & Hirabayashi, H. (2017). Comparison of predictability for human pharmacokinetics parameters among monkeys, rats, and chimeric mice with humanised liver. *Xenobiotica*, *47*(12), 1052–1063. <https://doi.org/10.1080/00498254.2016.1265160>
18. FDA approval package document Entocort (Budesonide Capsules) Company: AstraZeneca LP Application No.: 21-324 Approval Date: 10/2/2001 Pharmacology Review(s) P24 ; 2020 September 6. Available from <https://www.accessdata.fda.gov/drugsatfda_docs/nda/2001/21-324_Entocort_pharmr.pdf> [Website]
19. EMEA Cancidas (previously Caspofungin MSD) Assessment history Cancidas : EPAR - Scientific Discussion 2005 P10 ; 2020 September 6. Available from <https://www.ema.europa.eu/en/documents/scientific-discussion/cancidas-epar-scientific-discussion_en.pdf> [Website]
20. EMEA Doribax Public assessment report P18 ; 2020 September 6. Available from <https://www.ema.europa.eu/en/documents/assessment-report/doribax-epar-public-assessment-report_en.pdf> [Website]
21. Watanabe, T., Kusuhara, H., Watanabe, T., Debori, Y., Maeda, K., Kondo, T., Nakayama H, Horita S, Ogilvie BW, Parkinson A, Hu Z, Sugiyama Y. (2011). Prediction of the overall renal tubular secretion and hepatic clearance of anionic drugs and a renal drug-drug interaction involving organic anion transporter 3 in humans by in vitro uptake experiments. *Drug Metabolism and Disposition*, *39*(6), 1031–1038. <https://doi.org/10.1124/dmd.110.036129>
22. Naritomi, Y., Terashita, S., Kimura, S., Suzuki, A., Kagayama, A., & Sugiyama, Y. (2001). Prediction of human hepatic clearance from in vivo animal experiments and in vitro metabolic studies with liver microsomes from animals and humans. *Drug Metabolism and Disposition*, *29*(10), 1316–1324.
23. Deguchi, T, Watanabe, N, Kurihara, A, Igeta, K, Ikenaga, H, Fusegawa, K, Suzuki, N, Murata, S, Hirouchi, M, Furuta, Y, Iwasaki, M, Okazaki, O, Izumi, T. (2011). Human pharmacokinetic prediction of UDP-glucuronosyltransferase substrates with an animal scale-up approach. *Drug Metabolism and Disposition*, *39*(5), 820–829. <https://doi.org/10.1124/dmd.110.037457>
24. Iwatsubo, T., Hirota, N., Ooie, T., Suzuki, H., Shimada, N., Chiba, K., Ishizaki T, Green CE, Tyson CA, Sugiyama Y. (1997). Prediction of in vivo drug metabolism in the human liver from in vitro metabolism data. *Pharmacology and Therapeutics*. *73*(2), 147–171<https://doi.org/10.1016/S0163-7258(96)00184-2>
25. Brown, H. S., Griffin, M., & Houston, J. B. (2007). Evaluation of cryopreserved human hepatocytes as an alternative in vitro system to microsomes for the prediction of metabolic clearance. *Drug Metabolism and Disposition*, *35*(2), 293–301. <https://doi.org/10.1124/dmd.106.011569>
26. FDA approval package document Clinical Pharmacology and Biopharmaceutics Review NDA 205625 Fluticasone furoate P22 ; 2020 September 6. Available from <https://www.accessdata.fda.gov/drugsatfda_docs/nda/2014/205625Orig1s000ClinPharmR.pdf> [Website]
27. FDA approval package document Frova (Frovatriptan) Tablets Company: Elan Pharmaceuticals Application No.: 21-006 Approval Date: 11/8/01 Clinical Pharmacology Biopharmaceutics Review(s) P7 ; 2020 September 6. Available from <https://www.accessdata.fda.gov/drugsatfda_docs/nda/2001/21-006_Frova_biopharmr.pdf> [Website]
28. Sohlenius-Sternbeck, A. K., Afzelius, L., Prusis, P., Neelissen, J., Hoogstraate, J., Johansson, J., Floby, E, Bengtsson, A, Gissberg, O, Sternbeck, J, Petersson, C. (2010). Evaluation of the human prediction of clearance from hepatocyte and microsome intrinsic clearance for 52 drug compounds. *Xenobiotica*, *40*(9), 637–649. <https://doi.org/10.3109/00498254.2010.500407>
29. Walker, D. K., Abel, S., Comby, P., Muirhead, G. J., Nedderman, A. N. R., & Smith, D. A (2005). Species differences in the disposition of the CCR5 antagonist, UK-427,857, a new potential treatment for HIV. *Drug Metabolism and Disposition*, *33*(4), 587–595. <https://doi.org/10.1124/dmd.104.002626>
30. FDA approval package document Selzentry (maraviroc) 150 mg, and 300 mg tablets Company: Pfizer, Inc. NDA: 022128 Approval Date: 8/6/2007 Clinical Pharmacology Biopharmaceutics Review(s) ; 2020 September 6. Available from <https://www.accessdata.fda.gov/drugsatfda_docs/nda/2007/022128s000_ClinPharmR.pdf> [Website]
31. Moffat, Anthony C; Osselton, M David; Widdop, Brian; Watts, Jo Clarke's Analysis of Drugs and Poisons Fourth edition p1709
32. FDA approval package document Exelon (Rivastigmine Tartrate) Capsules Company: Novartis Pharmaceuticals Corporation Application No.: 20-823 Approval Date: 4/21/2000 Clinical Pharmacology Biopharmaceutics Review(s) P8 ; 2020 September 6. Available from <https://www.accessdata.fda.gov/drugsatfda_docs/nda/2000/20823_Exelon_biopharmr.pdf> [Website]
33. FDA approval package document Avandia (Rosiglitazone Maleate) Tablets Company: SmithKline Beecham Pharmaceuticals Application No.: 21-071 Approval Date: 5/25/1999 Clinical Pharmacology Biopharmaceutics Review(s) P177 ; 2020 September 6. Available from <https://www.accessdata.fda.gov/drugsatfda_docs/nda/99/21071_Avandia_biopharmr_P4.pdf> [Website]
34. Dingemanse, J., Clozel, M., & Giersbergen, P. L. M. Van. (2002). *Pharmacokinetics and pharmacodynamics of tezosentan , an intravenous dual endothelin receptor antagonist , following chronic infusion in healthy subjects*. 3(4), 355–362.
35. FDA approval package document Gabitril (Tiagabine HCl) Company: Cephalon Application No.: 20646 Approval Date: 9/30/1997 Pharmacology Review(s) P16 ; 2020 September 6. Available from <https://www.accessdata.fda.gov/drugsatfda_docs/nda/97/020646ap_gabitril_phrmr.pdf> [Website]
36. Watanabe, T., Kusuhara, H., Watanabe, T., Debori, Y., Maeda, K., Kondo, T., Nakayama, H, Horita, S, Ogilvie, BW, Parkinson, A, Hu Z, Sugiyama, Y. (2011). Prediction of the overall renal tubular secretion and hepatic clearance of anionic drugs and a renal drug-drug interaction involving organic anion transporter 3 in humans by in vitro uptake experiments. *Drug Metabolism and Disposition*, *39*(6), 1031–1038. <https://doi.org/10.1124/dmd.110.036129>
37. FDA approval package document Zometa (Zoledronic Acid) Injection Company: Novartis Pharmaceuticals Corporation HIGHLIGHTS OF PRESCRIBING INFORMATION ; 2020 September 6. Available from <https://www.accessdata.fda.gov/drugsatfda_docs/label/2014/021223s028lbl.pdf> [Website]
38. Poulin, P., & Theil, F. P. (2002). Prediction of pharmacokinetics prior to in vivo studies. II. Generic physiologically based pharmacokinetic models of drug disposition. *Journal of Pharmaceutical Sciences*, *91*(5), 1358–1370. <https://doi.org/10.1002/jps.10128>
39. Tenero, D. M., Bottorff, M. B., Given, B. D., Kramer, W. G., Affrime, M. B., Patrick, J. E., & Lalonde, R. L. (1989). Pharmacokinetics and pharmacodynamics of dilevalol. *Clinical Pharmacology and Therapeutics*. 46(6), 648–656. <https://doi.org/10.1038/clpt.1989.201>
40. Smith, D. A., Beaumont, K., Maurer, T. S., & Di, L. (2015). Volume of Distribution in Drug Design. *Journal of Medicinal Chemistry*, *58*(15), 5691–5698. <https://doi.org/10.1021/acs.jmedchem.5b00201>
41. FDA approval package document Ixempra (Ixabepilone) Injection Company: Bristol-Myers Squibb Co. Application No.: 022065 Approval Date: 10/16/2007 Clinical Pharmacology Biopharmaceutics Review(s) P15-16 ; 2020 September 6. Available from <https://www.accessdata.fda.gov/drugsatfda_docs/nda/2007/022065s000_ClinPharmR.pdf> [Website]
42. Skopp, G., Pötsch, L., Ganßmann, B., Aderjan, R., & Mattern, R. (1998). A preliminary study on the distribution of morphine and its glucuronides in the subcompartments of blood. *Journal of Analytical Toxicology*, *22*(4), 261–264. <https://doi.org/10.1093/jat/22.4.261>
43. Aymard, G., Warot, D., Démolis, P., Giudicelli, J. F., Lechat, P., Le Guern, M. E., Alquier, C., & Diquet, B. (2003). Comparative pharmacokinetics and pharmacodynamics of intravenous and oral nefopam in healthy volunteers. *Pharmacology and Toxicology*. *92*(6), 279–286. <https://doi.org/10.1034/j.1600-0773.2003.920605.x>
44. Calandre, E. P., Alferez, N., Hassanein, K., & Azarnoff, D. L. (1981). Methapyrilene kinetics and dynamics. *Clinical Pharmacology and Therapeutics*. 29(4), 527–532. <https://doi.org/10.1038/clpt.1981.74>
